# A prospective cohort study of soy product intake and stomach cancer death

**DOI:** 10.1038/sj.bjc.6600349

**Published:** 2002-07-15

**Authors:** C Nagata, N Takatsuka, N Kawakami, H Shimizu

**Affiliations:** Department of Public Health, Gifu University School of Medicine, 40 Tsukasa-machi, Gifu 500–8705, Japan

**Keywords:** soybeans, isoflavones, stomach cancer, mortality, diet

## Abstract

The relationship between intake of soy products and death from stomach cancer was examined in a community-based prospective study of Japanese men and women in Takayama, Japan. Over 7 years of follow-up, 121 deaths from stomach cancer (81 men and 40 women) occurred among 30 304 (13 880 men and 16 424 women) participants who were at least 35 years of age. Diet including the intake of soy products and isoflavones was assessed by a validated semiquantitative food–frequency questionnaire at the beginning of the study. In men, the highest compared to the lowest tertile of total soy product intake was significantly inversely associated with death from stomach cancer after controlling for covariates (hazard ratios=0.50; 95% confidence intervals (CIs) 0.26-0.93, *P* for trend=0.03). Decreased hazard ratios for the highest compared to the lowest tertiles of total soy product intake (hazard ratios=0.49; 95% CI 0.22–1.13) was observed in women, although this association was of marginal significance. These data suggest that soy intake may reduce the risk of death from stomach cancer.

*British Journal of Cancer* (2002) **87**, 31–36. doi:10.1038/sj.bjc.6600349 www.bjcancer.com

© 2002 Cancer Research UK

## 

The anti-cancer properties of soy isoflavones, i.e., genistein and daidzein, have been demonstrated in experimental studies (Adlercreutz *et al*, 1995). Interest focussed initially on the effects of soy isoflavones on hormone dependent cancers such as breast cancer and prostate cancer, mainly because of their ability to bind to the oestrogen receptor (Martin *et al*, 1978).

An inverse ecological correlation was reported between stomach cancer deaths in 47 Japanese prefectures and intake of soy products and isoflavones estimated from national nutritional survey data, raising the possibility that soy has a potential preventive effect against stomach cancer (Nagata, 2000). Certain analytic epidemiological studies on soy intake and stomach cancer, as reviewed by Messina *et al* (1994) and Wu *et al* (2000). However, soy intake in relation to stomach cancer was not a primary objective of these studies and only a limited number of soy-based items were covered and the association of stomach cancer with intake of isoflavones or soy products as a whole was not assessed.

We have investigated the relationship between soy intake and subsequent death from stomach cancer among Japanese men and women in a cohort study using a validated dietary questionnaire that listed various types of soy foods. Analysis was done separately for fermented and non-fermented soy products, because Wu *et al* (2000) presented the pooled odds ratio/relative risk estimates separately for these two catogories of soy products in a metaanalysis of previous studies on soy intake and stomach cancer.

## MATERIALS AND METHODS

The subjects were cohort members from the Takayama Study. The methology of the Takayama Study has been described previously (Shimizu, 1996). Briefly, the cohort included a total of 14 427 men and 17 125 women from Takayama City, Gifu, Japan, who were 35 years of age or older. Each participant completed a self-administered questionnaire in 1992 that was used to collect demographic and general information about smoking, alcohol, diet, exercise and medical and reproductive histories. The response rate was about 90%. Those who had smoked a total of 20 or more packs of cigarettes in their lifetime were defined as smokers.

Dietary history was assessed using a 169-item semiquantitative food-frequency questionnaire. Participants were asked to report the average frequency with which food was consumed in the previous year and the usual serving size of each food item. Nine food items for specific soy products and some others with soy products as ingredients were accounted for in order to estimate the total intake of soy products. Thus, total soy product intake was the sum of the intakes of tofu, miso, soybeans, natto, soymilk, okara, dried-tofu, deep-fried tofu, fried-tofu, fried tofu and minced vegetables/seaweed. The intake of foods and nutrients was estimated from the frequency of ingestion and portion size using the Japanese Standard Tables of Food Composition, 4th revised edition, published by the Science and Technology Agency of Japan. We also estimated the amounts for two main categories of soy products: fermented and non-fermented soy products. The fermented category included miso and natto. The other soy foods measured were non-fermented soy products. Isoflavone (daidzein plus genistein) contents are higher in fermented soy products; according to data summarised by Wakai *et al* (1999), isoflavone concentration per 100 g were 76.6 mg for miso and 67.4 mg for natto, while 24.1 mg for tofu and 36.2 mg for boiled soy beans. Salt is rich in miso, around 10 g per 100 g. The other soy products contain less than 1 g of salt per 100 g. Detailed information on the questionnaire concerning validity and reproducibility tests has been provided elsewhere (Shimizu *et al*, 1999; Nagata *et al*, 2001). For example, the Spearman correlation coefficients comparing estimates from this questionnaire and 12 daily diet records over 1 year were 0.75, 0.77 and 0.74 for soy intake in terms of the total amount (g) of soy products, daidzein, and genistein, respectively, in men. Corresponding values for women were 0.68, 0.63, and 0.65, respectively.

We excluded subjects who reported prevalent cancer (*n*=726; 186 men and 540 women) or a history of total/partial gastrectomy (*n*=399; 361 men and 161 women) at the baseline. Thus, the resulting baseline population was 30 304 (13 880 men and 16 424 women).

During 7 years of follow-up (1992–1999), deaths and their causes occurring in Takayama City were confirmed with data from the office of the National Vital Statistics. The Statistics and Information Department of the Japanese Ministry of Health and welfare obtain information on deaths and code the causes of death using the International Classification of Diseases (ICD). Permission to review the data regarding dates and causes of deaths was obtained from the Management and Coordination Agency, Japan. Stomach cancer was considered to be the underlying cause of death when the ICD-9 code was 151 or the ICD-10 code was C16. Information concerning subjects who moved away from Takayama City during the course of the study was obtained from the residential registers. During the study period, 648 (4.6%) men and 536 (3.3%) women moved out of Takayama City.

This study was approved by the local institutional review board.

To assess the association between soy intake and death from stomach cancer and to adjust for potential confounding factors, we computed hazard ratios (HRs) and their 95% confidence intervals (CIs) of stomach cancer death that occurred during the 7-year study by using Cox proportional hazard models. The length of follow-up was computed for each subject as the number of years elapsed from the start of the study (September 1, 1992) until the date of death due to stomach cancer, the date of death due to any causes other than stomach cancer, the date on which the person moved out of Takayama, or the end of the study (December 31, 1999). Food and nutrient intake was adjusted for total energy by using the method proposed by Willett (1990) and were converted into categorical variables based on the tertile of their distribution among the entire study population at the baseline. The lowest tertile was the reference category. Initial analysis examined the association between soy intake and death from stomach cancer after controlling for age and total energy. To examine whether the association of soy intake with stomach cancer death could be attributed to non-dietary or dietary variables other than soy product intake, the variables which were significantly associated with stomach cancer death were included in the models as covariates. The variables examined as potential confounders included marital status, body size, education, smoking and alcohol history, history of chronic diseases, use of medication including hormone replacement therapy, reproductive history and dietary components other than soy product intake. Tests for trend were performed on continuous variables using the median value on the categories. All the statistical analyses were performed using SAS programs (SAS Institute, Cary, NC, USA).

## RESULTS

During 208 951 person-years of follow-up over 7 years, 121 deaths (81 men and 40 women) occurred due to stomach cancer. Characteristics of participants by tertile of soy product intake are shown in Table 1

**Table 1:**
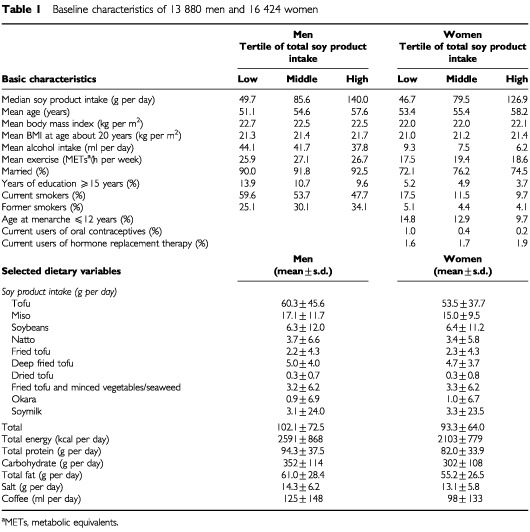
Baseline characteristics of 13 880 men and 16 424 women

.

In men, there was a statistically significant inverse association between the intake of total soy products and the rate of death from stomach cancer after controlling for age and total energy (Table 2

**Table 2:**
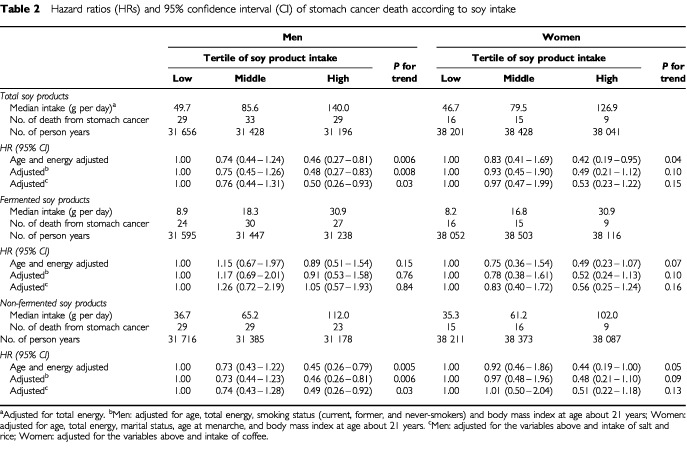
Hazard ratios (HRs) and 95% confidence interval (CI) of stomach cancer death according to soy intake

). The intake of non-fermented (but not fermented) soy products was significantly inversely associated with the death rate from stomach cancer. Among non-dietary factors, smoking status and body mass index at about 21 years of age were significantly associated with the rate of stomach cancer death after controlling for age; the HRs (95% CI) for current and former smokers compared to those who never smoked were 2.02 (1.07–3.80) and 1.18 (0.60–2.31), respectively. The HR (95% CI) for the second and highest compared to the lowest tertile of BMI at about 21 years of age were 2.05 (1.14–3.68) and 2.27 (1.22–4.20), respectively. After adjustment for these variables, the inverse associations for total soy product and non-fermented soy product intake remained statistically significant.

For men, of the other dietary components that included total protein, animal protein, vegetable protein, carbohydrate, carotene, crude fibre, salt, vitamin C, vitamin E, vegetables, fruits, rice, processed meats, fish and shellfish, dried fish, pickled vegetables, coffee, and green tea, salt intake was significantly inversely associated with stomach cancer mortality (HR for the highest compared to the lowest tertile=0.53; 95% CI (0.31–0.91)) and rice intake was significantly positively associated with stomach cancer mortality (HR for the highest compared to the lowest tertile=1.81; 95% CI 1.06–3.08) after controlling for age and total energy. Additional adjustment for the intake of salt and rice did not substantially alter the results.

Intake of fermented soy products was not associated with the death from stomach cancer. However, natto (but not miso) was associated with a decreased risk of stomach cancer death (HR=0.70 and 1.19 for the highest tertiles of natto and miso, respectively) after controlling for non-dietary and dietary covariates.

In women, the total amount of soy product intake was significantly inversely associated with death from stomach cancer after controlling for age and total energy (Table 2). Among other dietary and non-dietary variables measured, marital status, age at menarche, BMI at about 21 years of age, and intake of caffeinated coffee were significantly associated with the rate of death from stomach cancer after controlling for age: HR=2.15 for single compared to married; HRs=0.51, 0.41, and 0.18, for age at menarche 13–14, 15–16, and 17+ years compared to <=12 years, respectively; HR=3.36 and 2.16 for the second and the highest compared to the lowest tertile of BMI at about 20 years of age; HR=2.54 for daily compared to rare/never intake of caffeinated coffee. Simultaneous adjustment for these variables did not substantially alter the results. Both fermented and non-fermented soy product intake was inversely associated with the rate of death from stomach cancer, although the associations did not attain statistical significance. Miso intake was nonsignificantly but inversely associated with stomach cancer death in women (HR=0.55, 95% CI 0.23–1.34 for the highest tertile).

We re-analysed data excluding stomach cancer death that occurred during the first 4 years (*n*=38) in men. The HR estimates did not change substantially: HRs were 0.55, 1.15, and 0.54 for the highest compared to the lowest tertile of total, fermented, and non-fermented soy products, respectively.

## DISCUSSION

We observed a significantly reduced rate of death from stomach cancer in the highest compared with the lowest tertile of total soy product intake in men. In particular, a strong inverse association was observed for non-fermented soy products in terms of both the amount (g) and isoflavone intake (mg). A small number of deaths from stomach cancer in women resulted in a lack of statistical power to detect a significant association between soy intake and death from stomach cancer, but the magnitude of HR estimates for the intake of total soy products were similar to that observed for men. These data support the hypothesis that a high intake of soy is associated with decreased risk of stomach cancer.

To our knowledge, this is the first analytic epidemiologic study on stomach cancer to estimate soy and isoflavone intake using a validated questionnaire with a broad range of categories for soy products. Among previous prospective studies on diet and stomach cancer, six included one or two soy food items (Hirayama, 1984; Nomura *et al*, 1990; Kato *et al*, 1992; Inoue *et al*, 1996; Ahn, 1997; Galanis *et al*, 1998). In a Japanese study, Hirayama (1984) reported a significantly reduced risk of stomach cancer death associated with the daily intake of miso-soup (relative risk (RR)=0.74) compared to those who answered ‘never/rare’. No significant association between drinking miso soup and the risk of stomach cancer was reported in other four studies (Kato *et al*, 1992; Inoue *et al*, 1996; Galanis *et al*, 1998). Another soy product, tofu, was included in two studies. In a study of Japanese-American men reported by Nomura *et al* (1990), the RR of stomach cancer was 0.7 (95% CI 0.2–2.3) for those who ate tofu five times per week or more compared to those who ate it one time or less each week. Another study conducted in Korea showed a RR of 0.6 (95% CI 0.40–1.10) for the highest quartile of tofu intake (Ahn, 1997). Most of previous case–control studies included at most only two soy products (miso/soy bean paste or tofu/bean curd) (Wu *et al*, 2000).

Wu *et al* (2000) conducted a meta-analysis of the association of soy product intake with stomach cancer based on previous prospective and case–control studies. They calculated the pooled odds ratio/relative risk estimates separately for fermented and non-fermented soy products. The analysis of 14 studies excluding the study reported by Hirayama yielded an risk estimate of 1.26 (95% CI 1.11–1.43) in association with a high intake of fermented soy products. In contrast, the risk estimate was 0.72 (95% CI 0.63–0.82) for non-fermented soy product intake based on 10 studies. However, they also observed the similar patterns in risk of stomach cancer according to fermented soy product intake and salt intake in these studies. The association of stomach cancer risk with non-fermented soy products was similar to that with vegetables/fruit intake in these studies. Salt and vegetable/fruit intake was directly associated with stomach cancer risk. Therefore, they suggested the possibility that the reported associations for fermented soy products might be affected by the confounding effect of salt intake, and the reported associations for non-fermented soy products might be affected by confounding effects of vegetable/fruit intake. In almost all of the studies in their review, the possible confounding effects of salt, vegetables/fruits, and other dietary factors had not been considered in the soy product analysis. Our study also revealed a difference in the stomach cancer death rate for men based on their consumption of fermented and non-fermented soy products differently associated with the rate of stomach cancer death in men. However, the results on the association between non-fermented soy product intake and death from stomach cancer were not altered after controlling for vegetable or fruit intake in addition to the non-dietary and dietary covariates (for example, after additional adjustment for vegetable intake, HR=0.49 (95% CI 0.26–0.92) in men and HR=0.48 (95% CI 0.20–1.14) in women for the highest compared to the lowest tertile). Additional adjustment for total salt intake also did not alter the results for fermented soy products (HR=0.55 (95% CI 0.23–1.33) for the highest compared to the lowest tertile of intake in women; HR for men are shown in Table 2). These results suggest the negative association found between soy intake and stomach cancer death is not due to confounding with salt or vegetable/fruit intake. However, it is possible that other, non-measured factors, may contribute to some potential confounding. The fact that miso was negatively associated with stomach cancer death in women and that the other fermented soy food, natto, was also negatively associated with stomach cancer death in men, may suggest that the finding for miso in men is due to confounding factors.

Japan remains among the countries showing the highest mortality rates from stomach cancer in the world. The Japanese diet in Japan includes soybean-based foods that are rich in the isoflavones. It could be argued that our findings contradict these observations. The results from the present study imply that the high consumption of soy in Japan should not contribute to its high mortality rate from stomach cancer. We observed significantly positive association between the consumption of rice and stomach cancer mortality in men, and the association was marginally significant in women. High-starch diets have been hypothesised to be associated with stomach cancer (Ji *et al*, 1998). High rice consumption, which is common among the Japanese, may contribute to their high mortality rate from stomach cancer in Japan.

An inverse association of dietary soy with stomach cancer is biologically plausible. Isoflavones have been offered as the primary anticancer soy constituent. Genistein inhibited the growth of human gastric cancer cells (Yanagihara *et al*, 1993). Genistein attenuated gastric carcinogenesis by inducing increased apoptosis and lowering cell proliferation and angiogenesis of antral mucosa and gastric cancers (Tatsuta *et al*, 1999). A diet containing miso inhibited N-methyl-N′-nitro-N-nitrosoguanidine induced stomach tumours (Kim *et al*, 1985; Watanabe *et al*, 1999). It is also possible that other components in soy products might be etiologically important agents. Several laboratory studies have demonstrated that saponins as well as the Bowman–Birk Inhibitor isolated from soybeans have anti-carcinogenic properties (Von Hofe *et al*, 1991; Rao and Sung, 1995).

Use of population-based design is one of advantages of the present study. The response rate was high, and the number of people who were censored because they moved out of the city was small. We focused considerable attention on validating the method used for dietary assessment with the use of food records. Our questionnaire enabled us to estimate soy product intake quantitatively and to include careful adjustments for many potentially confounding factors.

There are several limitations in the present study. We examined the relation between soy intake and stomach cancer mortality, not incidence. Thus, our results reflect the potential effect of soy intake on stomach cancer incidence, survival, or both. If soy intake is related differently to incidence than it is to mortality, our results are potentially biased. For example, although the effect of gastric cancer screening on survival is not clear, there is a possibility that a high intake of soy might actually be associated with an increased incidence of stomach cancer combined with a higher participation rate in stomach cancer screening, which could lead to earlier diagnosis and better chances of survival. We obtained the information on history of participation in stomach cancer screening at the baseline but not during the follow-up period. Additional adjustment for history of participation in stomach cancer screening for 3 years prior to entry into the study did not alter the observed association between soy intake and risk of death from stomach cancer. For men, the HRs were 0.59 (95% CI 0.26–0.93), 1.05 (95% CI 0.56–1.96), 0.48 (95% CI 0.25–0.90), and 0.63 (95% CI 0.33–1.21) for the highest compared to the lowest tertiles of total, fermented and non-fermented soy products, and total isoflavones, respectively. The corresponding HRs for women were 0.52 (95% CI 0.23–1.20), 0.57 (95% CI 0.27–1.22), 0.58 (95% CI 0.26–1.30), and 0.54 (95% CI 0.23–1.27), respectively.

The prospective design of our study and exclusion of those with cancer and gastrectomy at the baseline lessen the possibility that disease status would bias the reporting of exposures. However, disease history obtained at the baseline was based on self-reporting. Those who reported a history of gastric ulcer may have been not informed that they actually had gastric cancer. When we re-analysed data excluding those who had reported a history of gastric ulcer (2041 men), however, the results were not altered substantially. The HRs (95% CI) were 0.50 (0.26–0.97), 1.13 (0.59–2.14), 0.47 (0.24–0.91), and 0.67 (0.34–1.29) for the highest as compared to the lowest tertiles of total, fermented and non-fermented soy products, and total isoflavones, respectively. We also tried to avoid the possibility that underlying stomach cancer should affect the diet by re-analysing data excluding deaths from stomach cancer that occurred during the first 4 years of the study.

The relatively short follow-up was also limitation. Fatal cases that progressed from diagnosis to death in 7 years or less may not have been representative of all stomach cancer cases, or even all stomach cancer deaths.

Adjustment for potential confounders did not modify the HR estimates. However, the high colinearity between various nutrients, foods, and food groups make it difficult to distinguish among the effects.

We could not obtain information on a history of infection with *Helicobacter pylori*, a major risk factor for stomach cancer. Intake of tofu was significantly inversely associated with *H. pylori* infection in a cross-sectional study of Japanese men (Shinchi *et al*, 1997). This study found no association of miso soup intake with *H. pylori* infection, but in another cross-sectional study of Japanese men (Tsugane *et al*, 1994), miso soup intake was significantly positively associated with *H. pylori* infection. High dose of genistein reduced *H. pylori* toxin-induced inflammatory cytokine expression (interleukin-8) (Ding *et al*, 1997). Genistein inhibited growth of *H. pylori* (Bae *et al*, 2001). Infection of *H. pylori* may be a potential confounding factor or a marker of certain confounding factor for the association between soy product intake and stomach cancer. It is also possible that *H. pylori* infection is an intermediate factor between soy product intake and stomach cancer.

Our questionnaire was designed to measure an individual's relative intake of food and nutrients rather than their absolute values. The data presented for food and nutrient intake may have been overestimated by the questionnaire. In the validity study, the estimates for soy product intake were about 20% higher for men and 40% higher for women when they were based on the questionnaire rather than on the estimates, which were based on 12 daily diet records over 1 year. The estimate of total energy was about 11% higher by questionnaire than by the diet records.

In summary, our data support for the notion that soy intake may be associated with risk of death from stomach cancer, either because of its beneficial effect on etiology or survival once stomach cancer occurs. Given the limitations mentioned above and the observational study design, this hypothesis remains tentative. In addition, the reason for a lack of an inverse association for miso, but not natto, is not clear. The potential effect of dietary soy on stomach cancer warrants further study.
